# Treatment Options for Class III Malocclusion in Growing Patients with Emphasis on Maxillary Protraction

**DOI:** 10.1155/2016/8105163

**Published:** 2016-04-10

**Authors:** Zeinab Azamian, Farinaz Shirban

**Affiliations:** Torabinejad Dental Research Center, Department of Orthodontics, School of Dentistry, Isfahan University of Medical Sciences, Isfahan 81746-73461, Iran

## Abstract

It is very difficult to diagnose and treat Class III malocclusion. This type of malocclusion involves a number of cranial base and maxillary and mandibular skeletal and dental compensation components. In Class III malocclusion originating from mandibular prognathism, orthodontic treatment in growing patients is not a good choice and in most cases orthognathic surgery is recommended after the end of growth. Approximately 30–40% of Class III patients exhibit some degree of maxillary deficiency; therefore, devices can be used for maxillary protraction for orthodontic treatment in early mixed dentition. In cases in which dental components are primarily responsible for Class III malocclusion, early therapeutic intervention is recommended. An electronic search was conducted using the Medline database (Entrez PubMed), the Cochrane Collaboration Oral Health Group Database of Clinical Trials, Science Direct, and Scopus. In this review article, we described the treatment options for Class III malocclusion in growing patient with an emphasis on maxillary protraction. It seems that the most important factor for treatment of Class III malocclusion in growing patient is case selection.

## 1. Introduction

Etiologic factors for Class III malocclusions include a wide spectrum of skeletal and dental compensation components [1]. The condition might be characterized by mandibular prognathism, maxillary retrognathism, retrusive mandibular dentition, protrusive maxillary dentition, and a combination of the above [2].

Clinically, Class III malocclusion is in two forms: (a) “pseudo or functional Class III,” due to an early interference with the muscular reflex of mandibular closure and (b) the “true Class III” [3].

The etiology of Class III malocclusion is multifactorial, with genetic, ethnic, environmental, and habitual components [4]. It was believed until 1970 that only the mandible is responsible for Class III malocclusion [5]; however, almost 30–40% of patients exhibit some degree of maxillary deficiency [6].

Different ethnic groups exhibit different prevalence rates of Class III, with different methods of classification being used. The prevalence rate was reported to be around 1–3% in the Caucasians and around 13-14% among the Chinese and Japanese [7–11]. In the Asian population the majority of patients exhibit midface deficiency [12]. It has been reported that more than 60% of Class III malocclusion cases are due to skeletal discrepancies [13].

Final and definitive diagnosis of skeletal Class III malocclusion is based on the following:

- Verification of the normal centric position with the habitual position.
- Presence or absence of a familial predisposition.
- Cephalometric parameters, including a decrease in SNA, negative ANB, mandibular protrusion, obtuse gonial angle, and great LAFH.
- Incisor relationship [14].

This study was undertaken to evaluate different types of devices used to correct Class III malocclusion in growing patients with an emphasis on devices used for maxillary protraction.

## 2. Search Strategy

A computerized search was carried out using the Medline database (Entrez PubMed, http://www.ncbi.nlm.nih.gov/), the Cochrane Collaboration Oral Health Group Database of Clinical Trials (http://www.cochrane.org/), Science Direct (http://www.sciencedirect.com/), and Scopus (http://www.scopus.com/) from 1956 to 2015 and from 20th February to 4th April 2015. The terms used included maxillary deficiency, Class III malocclusion, maxillary protraction, and bone anchors. During the preliminary search, 250 articles were selected based on the article titles and then, based on the aims of this study, 96 articles were evaluated.

## 3. Appliances

The options for correction of Class III malocclusion in growing patients consist of two principal categories: intraoral appliances and extraoral appliances (Table 1).

### 3.1. Intraoral Appliances

#### 3.1.1. Class III Elastics with Skeletal Anchorage

**Figure 1:**
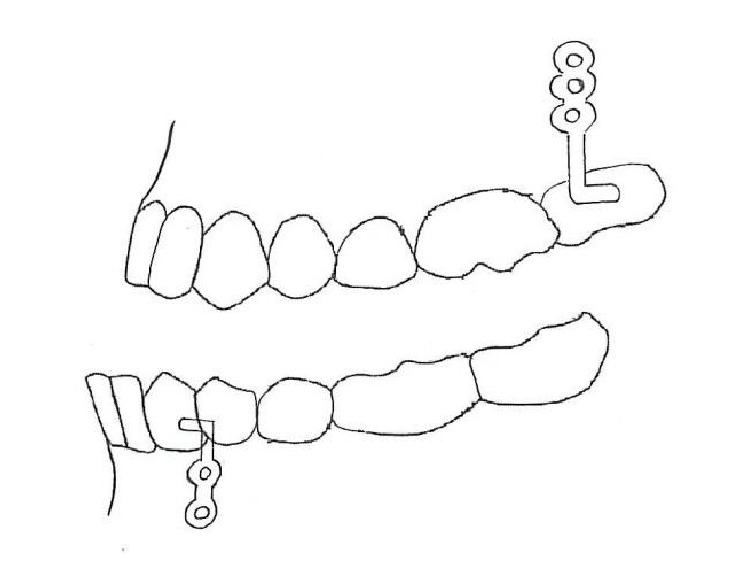
Class III elastic with skeletal anchorage.

Four miniplates are inserted in the left and right infrazygomatic crest of the maxillary buttress and between the lower left and right lateral incisors and canines (Figure 1). A mucoperiosteal flap is elevated and the miniplates are placed in the underlying bone by miniscrews. The extension of the plates perforates the attached gingiva and they are loaded three weeks later with Class III elastics [1].

#### 3.1.2. Bionator III

The reverse Bionator or Bionator III, a modified form of the traditional Bionator, is used in the treatment of Class III malocclusion cases. The modified Balters' Bionator III [15] exhibits differences from the original version, with deeper and wider lingual wings, acrylic vestibular lateral shields extending deep into the upper fornix, upper labial buttons, and upper incisor inclined plane. The construction bite is normally taken by gentle repositioning of the mandible in the centric relation. Patients are expected to wear such an appliance for a minimum of 22 hours a day [16].

#### 3.1.3. Frankel III Functional Appliance

Frankel III functional appliance is made while the mandible is positioned posteriorly. It has pads to stretch the upper lip and periosteum forward that stimulates forward growth of maxilla [17].

#### 3.1.4. Eschler Appliance

**Figure 2:**
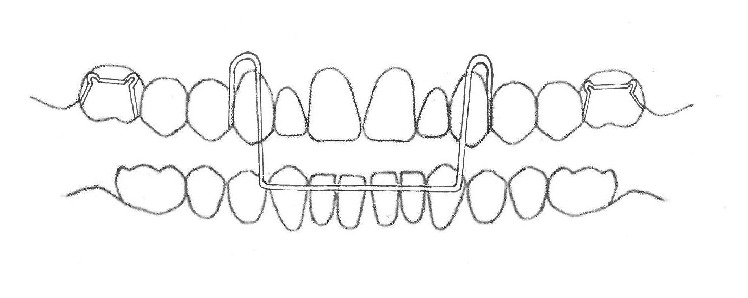
Eschler appliance.

The Eschler appliance consists of 3 parts. The first part is a retention component such as Adams clasps for molars and intermolar auxiliary clasps for deciduous teeth and premolars. The second part is an Eschler labial bow made of a 0.9 mm wire (Figure 2). The third part is occlusal bite raising, made of acrylic resin measuring 2-3 mm in thickness. An expansion screw or spring can be added for some specific purposes [18].

#### 3.1.5. Double-Plate Appliance

**Figure 3:**
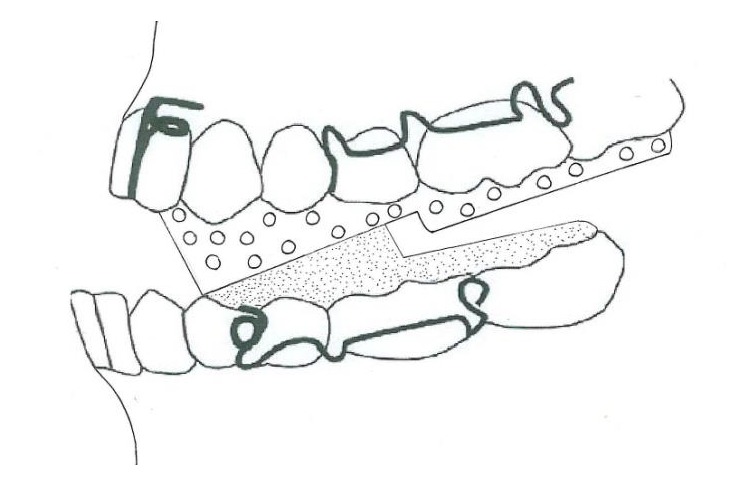
Double-plate appliance.

The double-plate appliance is an intraoral appliance containing angulated acrylic blocks, with an acrylic segment that contacts the lingual surfaces of lower incisors in order to prevent their retraction (Figure 3). This appliance is used with a face mask [19].

#### 3.1.6. Tandem Appliance

**Figure 4:**
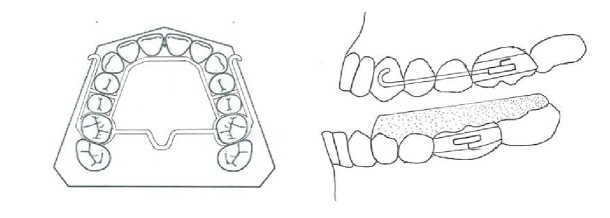
Tandem appliance.

The Tandem appliances are made up of three components. The upper appliance is fixed, with bands on deciduous second molars, a transpalatal arch, and palatal expansion arms and buccal arms for elastic traction. The lower appliance has bands on deciduous second molars, a lingual holding arch, a fixed bite plane for posterior occlusal coverage, and buccal face bow tubes (Figure 4). The outer bow of the headgear face bow has been modified to engage elastics and is inserted into the lower tubes [20].

### 3.2. Extraoral Appliances

#### 3.2.1. Chin Cap

Chin cap is a useful appliance in growing patients that exhibit mandibular prognathism and short lower facial height. It has been shown that chin cap redirects mandibular growth, rotates the mandible backward, retards mandibular growth, and remodels the mandible [21]. It also increases the anterior facial height. It is particularly more useful for Asian children compared to Caucasians, which is attributed to their shorter facial height and greater protrusion of lower incisors, rather than to differences in their response to treatment [17].

#### 3.2.2. Headgear for Mandibular Arch

Baccetti et al. [22] and Rey et al. [23] used the mandibular cervical headgear in growing Class III patients exhibiting mandibular prognathism. This treatment option results in distalization of mandibular molars and redirection of mandibular growth.

#### 3.2.3. Face Mask

Orthopedic protraction of maxilla in Class III patients exhibiting maxillary retrusion and meso- or brachyfacial patterns proved effective [24, 25]. The most effective appliance in such cases is a face mask. However, there are some limitations in the use of a face mask, including patient compliance problems, dentoalveolar effect, limited protraction of maxilla (2-3 mm in 9–12 months), and the possibility of relapse as a result of mandibular growth [26–30].

Face masks have various clinical applications. The clinician may choose a Petit face mask or a Delaire type as an extraoral part of the appliance, opt for skeletal anchorage versus dental anchorage, or choose advancement with expansion in contrast to advancement without expansion. Here, we are going to review use of face masks in dental clinics.

Delaire face mask is commonly used for protraction of maxilla. The chin and forehead are used for extraoral anchorage [31]. This appliance might interfere with sleep or wearing eyeglasses [17]. Petit modified the Delaire face mask in 1983, incorporating a forehead and a chin pad that were connected with a heavy steel rod [32].

#### 3.2.4. Protraction of Maxilla with Expansion and without Expansion

Use of rapid maxillary expansion (RME) has been recommended for protraction of maxilla. Some authors believe that expansion will disarticulate maxilla and initiate cellular response [33–35]. The appliance in the maxillary arch is a bonded or banded maxillary expander. The patient activates the expander once or twice a day until the desired transverse relationship is achieved [1]. Another protocol is the use of alternate rapid maxillary expansions and constrictions (Alt-RAMEC). Activation of expansion/constriction is 0.5 mm daily [36] to disarticulate the suture without overexpansion [37, 38]. However, a meta-analysis found that protraction was the same with or without expansion [29].

#### 3.2.5. Face Mask with Dental Anchorage

A routine protocol for face mask therapy is application of force to a removable appliance in the maxilla. There is consensus over application of force at 30° angulation to the occlusal plane for minimum unwanted rotation of the maxilla. Forces of 300–600 g on each side are favorable. The skeletal results obtained with different amounts of force (300–500 g) are similar, resulting in 3° increase in SNA [39].

#### 3.2.6. Protraction Face Mask and Reverse Twin Block

Early treatment of Class III malocclusions with protraction face mask and reverse twin block (PFM and RTB) might be effective. The remaining growth will influence the long-term stability of these treatments [40].

#### 3.2.7. Face Mask with Skeletal Anchorage: Bone Anchor Maxillary Protraction (BAMP)

*(1) Face Mask with a Titanium Screw.* Titanium screws have been successfully used as skeletal anchorage [41]. These screws do not require latency time for osseointegration, and treatment can be instituted immediately after insertion. In a case report, a lag titanium screw was applied as skeletal anchorage for protraction of maxilla. 800 g force per side was applied at a 30° angle relative to the occlusal plane. The anterior nasal spine was advanced approximately 3 mm anteriorly, with stable improvement after a year [42].

**Figure 5:**
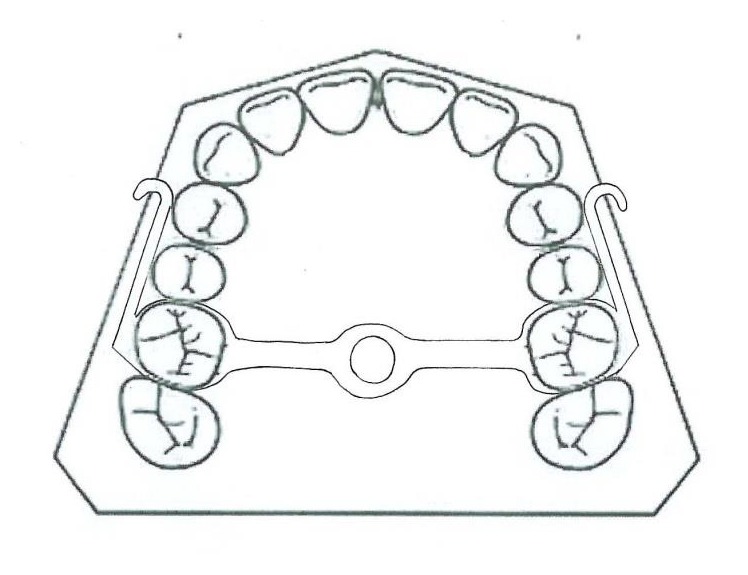
Face mask with skeletal anchorage.

*(2) Face Mask with Onplant.* In 1995 Block and Hoffman applied onplant as an anchorage for orthodontic purposes in animals [43]. The onplants were reported to tolerate forces up to 300 g. In a different study onplants were used for application of force to the maxilla. Subsequent to a surgical operation for insertion of onplants (7.7 mm hexagonal onplants) near the molar area (Figure 5), a vacuum-formed stent was used for 10 days. Osseointegration occurred over a period of 3-4 months. Then a 400 g force per side was transmitted to the hooks in the premolar area in the maxillary fixed appliance. The onplants, as a reference point, moved 2.9 mm horizontally and 2.9 mm vertically over a 12-month period [44].

*(3) Face Mask with Osseointegrated Implants*. The first clinical use of titanium implants as an anchorage for maxillary protraction occurred in an animal study. These Brånemark implants withstood 600 g force per side and an 8 mm advancement of the maxilla was achieved [45]. In a different study, implants were used in the zygomatic process of the maxilla and a 400 g force per side was applied, resulting in a 4 mm advancement of the maxilla [46].

*(4) Face Mask to an Ankylosed Primary Canine*. Use of an intentionally ankylosed tooth is a proper technique for direct transmission of force for protraction of the maxilla. However, such teeth undergo resorption as their permanent successors erupt, restricting use of ankylosed teeth to young patients [47–49].

#### 3.2.8. Corticotomy-Assisted Maxillary Protraction

Low-angle Class III patients who exhibit severe retrognathism of the maxilla, patients who have lost chance of orthopedic correction, and patients who refuse to undergo orthognathic surgeries are candidates for corticotomy-assisted maxillary protraction [50]. Sutural distraction osteogenesis versus osteotomy distraction osteogenesis for protraction of midface has already been used. Lefort III fractures have been used in the zygomaticofrontal suture. Distraction has been carried out with the use of heavy elastics [51].

Rachmiel et al. in 1999 [52] and Samchukov in 2001 [53] reported patients treated by an incomplete Lefort I osteotomy followed by face mask protraction. They reported 5–9 mm of maxillary protraction. In such a treatment modality a face mask is used for 5–7 days after surgery and a 1700–2000 g force is applied. Significant relapse of maxillary advancement was detected in a 6-year follow-up. However, well-preserved dental relationship was reported [54].

## 4. Discussion

Orthopedic treatments might prove effective in children with Class III malocclusion in the short term [55]. Several appliances are used for early treatment of skeletal Class III, including Bionator [15], Frankel (FR-III) [17], chin cup [21], double-plate appliance [19], Eschler appliance “progenic appliance” [18], and protraction face mask. Orthopedic protraction of the maxilla is a popular treatment modality, with some limitations, including problems with patient compliance, limited protraction of the maxilla (2-3 mm in 9–12 months), unwanted dentoalveolar effects, and the possibility of relapse as a result of late mandibular growth [28, 29, 56].

Face mask therapy is effective in Class III, maxillary-deficient, deep-bite patients, and all the treated patients exhibit positive overjet after treatment. In a study, after face mask therapy, the maxilla continued to grow in the anterior direction in an amount equal to untreated Class III patients but less than that in untreated Class I patients; mandibular growth was similar in all the groups [57].

### 4.1. Age of Intervention (Face Mask Therapy)

An important factor determining the success of treatment for Class III patients is treatment timing. It has been recommended that face mask therapy should be initiated at 6–8 years of age after eruption of maxillary permanent first molar and incisors, that is, early mixed dentition [20, 39, 58, 59]. However, maxillary protraction with bone anchors and Class III elastics has been reported to be successful in the late mixed or permanent dentition phases [1].

### 4.2. Maxillary Protraction with or without Maxillary Expansion?

In addition, rapid maxillary expansion (RME) has been recommended as a routine component of treatment for correction of Class III malocclusion, even in the absence of maxillary constriction because it disarticulates the maxilla and gives rise to cellular responses in the circummaxillary sutures, bringing about a more positive reaction to protraction forces [34, 35, 60]. Nevertheless, when used to enhance anterior movement of the maxilla during face mask therapy, preliminary RME does not appear to exert any effect on the efficacy of orthopedic treatment [61]. There are reports that use of RME alone might not properly disarticulate circummaxillary sutures and it might be better dealt with by Alt-RAMEC [36, 62, 63]. A meta-analysis showed similar results for protraction with or without expansion [29].

### 4.3. Treatment Outcome for Face Mask Therapy with Dental or Skeletal Anchorage

Cephalometric analyses have shown skeletal and dentoalveolar changes in face mask therapy. Skeletal changes include maxillary protrusive movement (SNA, N-perpA) and downward and backward rotation of the mandible (SNGoGN, SNGn, and LAFH), with a decrease in prognathism severity (SNB). Such changes induce favorable changes in the facial profile. Dentoalveolar changes mainly consist of linguoversion of mandibular incisors and labial inclination of maxillary incisors [64]. The forces used for maxillary protraction are usually applied to maxillary teeth. Therefore, there might be a significant mesial migration of maxillary teeth, possibly resulting in severe anterior crowding and in a decrease in the orthopedic effects of treatment, which might cause problems [65, 66]. It has been demonstrated that the mandibular plane angle increases significantly during protraction face mask therapy [67–69]. Some of the complications associated with dental anchorage are resolved by skeletal anchorage. Recently, osseointegrated implants, titanium screws, onplants, and miniplates have been used as stable anchorage for maxillary protraction [41, 46, 56, 70].

The greatest disadvantage of palatal osseointegrated implants is that they can only be placed on the palate and are indicated for moving maxillary teeth. A palatal implant costs much higher than a miniscrew and miniplate and requires an osseointegration period [71].

Temporary anchorage devices for maxillary protraction have become very popular in recent years [1, 42, 44, 46, 72].

A study showed an improvement in molar relation, which was not influenced by dental inclination and there was no significant amount of lingual inclination of the lower incisors in the BAMP group [1].

The proclination of mandibular incisors might be explained by the new posture of the tongue that acts on these incisors after correction of anterior crossbite [56, 73].

In Class III cases that are treated with skeletal anchorage, the amount of maxillary protraction might vary from 3.0 to 5.6 mm [36, 73–75]. The BAMP protocol can cause significantly larger maxillary advancement compared to the RME/FM therapy. BAMP results in 2.3–3 mm more maxillary protraction compared to face mask or rapid maxillary expansion [1]. BAMP protocol results in fewer vertical changes. Furthermore, these patients do not exhibit clockwise rotation of the mandible or dental compensation [1, 76]. A 3.82 mm forward movement of the nose tip was reported as a result of the BAMP protocol [77]. The upper lip and lip sulcus also moved forward, and the soft tissue B point and pogonion moved backward during the protraction period, indicating improvements in the soft tissue profile in line with the underlying skeletal components during the protraction procedure [56, 78, 79].

It has been reported that it is difficult to control the vertical growth and lower anterior facial height increases to some extent during maxillary protraction, with both dental and bone anchorage [1, 26, 34, 80]. The low mandibular plane angle group exhibited a greater maxillary forward displacement and a larger increase in the maxillary body compared to the high mandibular plane angle group [81]. The craniomaxillary complex in the dental anchorage model is displaced forward along with rotation, and the amount of this rotation decreases gradually with an increase in the angle between the force vector and occlusal plane from 0 to 30 degrees. However, the craniomaxillary complex in the bone anchorage model is displaced forward along with rotation, and the rotation degree decreases gradually with an increase in the angle from 0 to 20 [82].

A cephalometric analysis of pharyngeal airway showed no significant changes in the oro- and nasopharyngeal sagittal airway dimensions in the face mask + bite block therapy group compared to the untreated Class III subjects [83].

Ghiz et al. [84] reported that four variables were significant in predicting successful treatment outcomes: (1) the position of the condyle relative to the cranial base; (2) ramus length; (3) mandibular length; and (4) gonial angle [85]. Three pretreatment cephalometric variables exhibited the highest predictive power in terms of discriminating gonial angle, nasion-A-pogonion angle, and ramus plane-to-sella-nasion angle. Patients exhibiting larger values for these three measurements before treatment were categorized as the unstable group at the end of the observation period. Orthopedic treatment of Class III malocclusion might give rise to more favorable craniofacial adaptations when a patient's pretreatment cephalometric analyses reveal a short mandibular ramus (i.e., decreased posterior facial height) and a low mandibular plane angle [30].

### 4.4. Skeletal Analysis of Treatment Outcomes

An approach is to make use of a 3D skeletal color map of the superimposition on the anterior cranial base. The superimposition and semitransparent overlays show that bone-anchored maxillary protraction growth and treatment response lead to bone apposition at the anterior eminence of the TMJ, which correlates well with the posterior displacement of the anterior surface of the condyle, and the bone resorption of the posterior wall of the articular eminence correlates well with the posterior displacement of the posterior surface of the condyle. Mandibular shape, rather than the mandibular size, is under the influence of continuous intermaxillary traction [14]. Forward movement of the maxilla might be registered at the posterior nasal spine and at pterygomaxillary fissure points [72].

Another method is to use the patient's lateral cephalograms, which can be extracted from CBCT, in order to carry out TPS analysis [86]. TPS analysis deforms one landmark configuration into another, indicating that this change in shape is the deformation of a grid, at the same time making statistical comparisons possible. TPS has specific cephalometric indications to demonstrate differences in shape as a result of orthodontic treatment techniques or growth-related changes. In fact, TPS analysis has been used to study growth changes in treated and untreated subjects with different types of malocclusion [87, 88].

### 4.5. Retention and Follow-Up

In previous studies retention has been recommended after overjet and overbite correction from three months to two years during the night [24, 89, 90]. Long-term follow-ups of maxillary protraction indicate a 25–33% chance of relapse to negative overjet after the completion of mandibular growth [30, 67, 91, 92]. It was concluded that relapse to a Class III pattern primarily results from mandibular growth rather than a relapse in the maxilla [50, 66]. In another study, patients with larger values for the inclination of the mandibular ramus to the mandibular body (gonial angle) before treatment exhibited a higher probability of relapse at the end of the observation period [30, 93, 94].

## 5. Conclusion

An important factor for treatment of Class III malocclusion in growing patient is the origin of malocclusion. The skeletal or dental origin of the malocclusion and in skeletal Class III malocclusions mandibular prognathism or maxillary deficiency are important for choosing early intervention and selection of the appliance for treatment. All appliances described in this paper can be useful when the clinicians use them in correct manner.

## Figures and Tables

**Table 1:** Appliances for correction of class III malocclusion in growing patients.

Intraoral appliances	Fixed	Class III elastic with skeletal anchorage [1]	Skeletal effect
	Removable	Modified Balters' Bionator III [15]	Dental effect
		Frankel III [17]	Skeletal/dental effect
		Reverse twin block [40]	Dental effect
		Eschler/progenic appliance (removable mandible retractor) [18]	Dental effect
		Double-piece corrector [19]	Dental effect
		Tandem appliance [20]	Skeletal/dental

Extraoral appliances	Chin cap [21]		Skeletal
	Face mask [24, 25]		Skeletal
	Headgear for mandibular arch [22, 23]		Skeletal/dental
